# Contact Screening for Healthcare Workers Exposed to Patients with COVID-19

**DOI:** 10.3390/ijerph17239082

**Published:** 2020-12-05

**Authors:** Luca Coppeta, Giuseppina Somma, Lorenzo Ippoliti, Cristiana Ferrari, Iacopo D’Alessandro, Antonio Pietroiusti, Marco Trabucco Aurilio

**Affiliations:** 1 Department of Biomedicine and Prevention, University of Rome Tor Vergata, 00133 Rome, Italy; giuseppina.somma@ptvonline.it (G.S.); pietroiu@uniroma2.it (A.P.); 2 School of Occupational Medicine, University of Rome Tor Vergata, 00133 Rome, Italy; ippoliti.lo@libero.it (L.I.); cristianaferrari.md@gmail.com (C.F.); iacopodalessandro@libero.it (I.D.); 3 Department of Medicine and Health Sciences “V. Tiberio”, University of Molise, 86100 Campobasso, Italy; marco.trabuccoaurilio@unimol.it

**Keywords:** SARS-CoV-2, COVID-19, contact screening, HCWs

## Abstract

In China and Italy, many cases of coronavirus disease 2019 (COVID-19) have occurred among healthcare workers (HCWs). Prompt identification, isolation and contact tracing of COVID-19 cases are key elements in controlling the COVID-19 pandemic. The aim of this study was to evaluate the rate of Severe Acute Respiratory Syndrome Coronavirus 2 (SARS-CoV-2) infection among HCWs exposed to patients with COVID-19 in relation to the main determinants of exposure. To assess the risk of exposure, we performed active symptom monitoring in 1006 HCWs identified as contacts of COVID19 cases. The presence of symptoms was statistically associated with a positive nasopharyngeal swab result. Only one subject was asymptomatic at the time of positive test. These data suggest that clinical history may help in the selection of subjects to be investigated by means of reverse transcriptase-polymerase chain reaction (RT-PCR) in the case of a shortage of diagnostic resources. We found that close contact (within 2 m for 15 min or more) was not statistically related to contagion. Regarding the use of personal protective equipment (PPE), only the use of facial masks was inversely related to the chance of becoming infected (*p* < 0.01). In conclusion, our data show that unprotected contacts between HCWs should be considered a major route of HCW contagion, suggesting that the use of facial masks should be implemented even in settings where known patients with COVID-19 are not present.

## 1. Introduction

While Severe Acute Respiratory Syndrome Coronavirus 2 (SARS-CoV-2) continues to spread worldwide, the strain on the global healthcare system is to escalate because of the potentially overwhelming burden of illnesses that stresses the health system capacity and also because of the adverse effects on healthcare workers (HCWs), including the risk of infection [1].

As a matter of fact, a large proportion of reported cases of coronavirus disease 2019 (COVID-19) in China and Italy, especially in the initial stage of the outbreaks, have occurred among HCWs, infected by both patients and colleagues [2]. The inadequate protection devices and the still unknown characteristics of the virus, at the beginning of the epidemic, contributed to the contagion of HCWs. Infectious diseases among HCWs may have important consequences in stressing the health system capacity. In fact, in addition to further compromising the health of hospitalized patients, the spread of the disease to colleagues may result in a reduction in healthcare personnel because of the need to adopt quarantine measures for the close contact cases within the staff. Therefore, infection prevention and control (IPC) practices are of critical importance in controlling the COVID-19 pandemic, protecting the functioning of healthcare services and mitigating the impact on vulnerable populations. Based on the experience acquired during the 2002 Severe Acute Respiratory Syndrome (SARS) outbreak, more emphasis should be placed on the protection of HCWs, considering that 1725 of front-line HCWs were infected by SARS [3].

In the current COVID-19 epidemic, the infection of medical and nursing personnel represents a common occurrence, ever since the first 15 affected cases were reported in Wuhan, when the disease was still confined in China. It was estimated that 1716 Chinese HCWs were infected by COVID-19 until 11 February 2020 [4]. Sadly, on 24th February, the National Health Commission of the People’s Republic of China (NHCPRC) reported in a press conference of WHO-China Joint Mission on COVID-19 that 3387 healthcare workers had contracted COVID-19, and that 22 (0.6%) had died [5].

Inadequate personal protection of HCWs at the beginning of the epidemic, long-time exposure to a high number of infected patients, shortage of personal protective equipment (PPE), pressure of early treatment, work intensity and prolonged shifts, have been reported as major causes of HCW involvement [6].

Prompt identification, isolation and contact tracing are common interventions to control infectious disease outbreaks: they can be effective but might require intensive public health effort and cooperation to reach and monitor all contacts. In the case of the COVID-19 epidemic, it might be useful to prioritize HCWs having had contact with people with confirmed COVID-19 [7].

According to the European Centre for Disease Prevention and Control (ECDC) definition, a contact with a COVID-19 case is a person not currently presenting symptoms, who has, or may have, been in contact with a patient with SARS-CoV-2; the associated risk of infection depends on the level of exposure, which determines the type of monitoring [8].

The main advantage of contact tracing during infectious disease outbreaks is that it can identify potentially infected individuals before the onset of symptoms, and, in the case of early identification, it may prevent subsequent transmission from secondary cases.

Case investigation, contact tracing and risk assessment, with an appropriate implementation of work restrictions for the potentially exposed HCWs, remain the recommended strategy to identify and reduce the risk of transmission of COVID-19 among HCWs, patients and others [2].

The aim of our study was to evaluate the rate of SARS-CoV-2 infection among HCWs exposed to COVID-19 cases, in relation to the main determinants of exposure.

## 2. Materials and Methods

To assess the risk of SARS-CoV-2 transmission, we interviewed the HCWs identified as contacts of COVID-19, according to the ECDC definition.

In particular, we evaluated (for each HCW involved in the screening): the distance from the infected subject (more or less than 2 m), the duration of exposure (more or less than 15 min), the kind of assistance provided in the case of hospitalized patients and the use of adequate personal protective equipment such as masks (medical, FFP2, FFP3), gloves and eye protection devices (goggles, face shields).

All contacts were interviewed about their exposure to determine any possible need for work restrictions. We considered exposed: HCWs not wearing appropriate PPE who reported having had a prolonged close contact with patients with COVID-19 (wearing or not a facemask), and all the medical staff not wearing appropriate PPE (e.g., wearing a medical mask when carrying out aerosol generating procedures on patients with COVID-19). Exposed HCWs were included in an active surveillance program and contacted daily to monitor the possible occurrence of fever (HCWs were asked to check their temperature twice a day), respiratory symptoms (e.g., cough, shortness of breath, sore throat) or other symptoms (anosmia, asthenia, myalgia, headache, digestive symptoms). Moreover, they were asked to immediately self-isolate and contact our unit (the Department of Occupational Health), if any of the above-mentioned symptoms developed during active surveillance. In this case, HCWs promptly underwent a nasopharyngeal swab for the detection of SARS CoV-2 RNA.

The removal of the nasopharyngeal and oropharyngeal swab is a procedure that consists in the removal of mucus that lines the superficial cells of the mucous membrane of the nasopharynx or oropharynx, using a swab. The oropharyngeal swab is performed first.

The patient is asked to keep their mouth wide open and to stick out their tongue. The tip of the swab reaches the pharyngeal cable along the area between the tonsil pillars and behind the uvula and rubs the tonsil area. It is important to prevent the swab being contaminated with saliva, avoiding touching the tongue, cheeks or dental arches.

Subsequently, the pharyngeal swab is immersed in the solution of the test tube and rotated keeping it immersed to disperse the samples collected in the liquid of the test tube as much as possible.

Then, the pharyngeal swab is removed from the test tube, placed in the decontaminant solution for inactivating the viral load and then packed away in a rigid bin.

At the end of the procedure, the sampler invites the patient to wear a mask and to carry out hand hygiene with hydroalcoholic solution and then to leave the room, after explaining how to collect the results.

The Seegene AllplexTM2019-nCov Assay was used to test oropharyngeal and nasopharyngeal swabs in line with the manufacturer’s protocols. The Allplex™ 2019-nCoV Assay is an in vitro diagnostic (IVD) real-time reverse transcriptase polymerase chain reaction (RT-PCR) test intended for the qualitative detection of SARS-CoV-2 viral nucleic acids. 

The automated nucleic acid extraction system isolates and purifies nucleic acids from 300 μL of specimens. An amount of 10 μL of Internal Control (RP-V IC) must be added before the extraction. An amount of 8 μL of purified nucleic acid is reverse transcribed using 5X Real-time One-step Buffer/Real-time One-step Enzyme into cDNA, which is then subsequently amplified in a CFX96TM and CFX96 Touch™ Real-Time PCR Detection System.

During the process, the probe anneals to a specific target sequence located between the forward and reverse primers. During the extension phase of the PCR cycle, the 5′ nuclease activity of Taq polymerase degrades the probe, causing the reporter dye to separate from the quencher dye, generating a fluorescent signal. With each cycle, additional reporter dye molecules are cleaved from their respective probes, increasing the fluorescence intensity. RT-qPCR was run on a CFX96TMDx platform (Bio-Rad Laboratories, Inc., Hercules, CA, USA) and subsequently interpreted by Seegene’s Viewer Software.

The Seegene AllplexTM2019-nCoV Assay identifies the virus by multiplex real-time PCR targeting three viral genes (E, RdRP and N), thus complying with international validated testing protocols. The ”Seegene viewer” shows whether the exported data are 2019-nCoV Detected, Presumptive positive or Negative for easy retrieval of the result by the user.

We then conducted an active symptoms monitoring of the 1006 contacts. HCWs were asked daily (for 14 days from the last known contact with a person with a diagnosis of COVID-19) by means of a phone call, about the onset of sore throat, cough, fever, dyspnea, fatigue, myalgia, anosmia and/or digestive symptoms.

Data were entered into an Excel spreadsheet, and statistical analysis was performed using SPSS Software (release 11).

Fisher’s exact test was used to determine whether there was a significant association between case investigation characteristics and results of the nasopharyngeal swab.

For each subject, we collected age, gender, characteristics of exposure, rate of symptomatic subjects, time elapsed from exposure and the onset of symptoms. The rate of positive swab results was then calculated.

## 3. Results

We evaluated the clinical records of 1006 HCWs (345 male and 661 female).

The mean age of the HCWs included in the study was 42 years old (range: 23–69 years).

We found 12 HCWs with positive swab (1.19%). Time elapsed from the last exposure and the positive RT-PCR result ranged from 3 to 13 days (mean 6.7 days). Most of the operators showing positive swab result were females (9/12), whereas male gender (3/12) was less represented.

Among infected HCWs, only one was taking medication for hypertension. No other relevant comorbidities were found.

Typical COVID-19 symptoms were present in 11 out of 12 (92%) HCWs with a positive swab test result (for SARS-CoV-2) and in 331 out of 994 (33.3%) HCWs with a negative swab (*p* < 0.01). Among symptoms: fever, anosmia, asthenia, myalgia, headache, fever and cough were statistically related to a positive swab result.

We found that neither a close contact with a COVID-19 case (within 2 m) nor a long exposure time (for a total of 15 min or more) were statistically related to a positive swab result, after performing Fisher’s exact test analysis. Regarding the use of PPE, only the use of masks was found to be protective from contagion (*p* < 0.01).

Most of the positive subjects had had an unprotected contact with a colleague (diagnosed with COVID-19) rather than with a hospitalized patient (8 vs. 12).

## 4. Discussion

Mass testing of healthcare workers (HCWs) exposed to COVID-19 cases, regardless of symptoms, has the aim both to mitigate workforce shortage due to unnecessary quarantine and to avoid the spread from asymptomatic HCWs, thus protecting both their colleagues and patients.

The presence of asymptomatic cases of COVID-19 is well recognized, and their number is relevant, up to 19.9% of all cases [9]. The COVID-19 infectiousness model suggests that in 44% of secondary cases the contagion occurred during the presymptomatic stage of the infection in the index case [10]. Routine and post-exposure testing of HCWs exposed to an active COVID-19 case might mitigate nosocomial transmission of Sars-Cov-2: a retrospective survey carried out in Wuhan reported that 41% of infected patients were likely to have been infected in the hospital setting [11].

Our investigation adds substantial knowledge to the evaluation of post-exposure screening of HCWs in the case of contact with a patient or colleague with COVID-19.

Results of our study show that the attack rate among HCWs exposed to unrecognized patients with COVID-19 is 2.4%. From our data, all subjects with a positive molecular test have the potential to get sick until the tenth day from the last exposure to a person with COVID-19, and most of them (8 out of 12) during the first seven days. Therefore, we can conclude that the median time from the contact with a COVID-19 case to the development of symptom onset may be up to ten days.

Self-reported symptoms were significantly associated with a positive swab result, and only one subject was asymptomatic on the day he was tested. Moreover, this subject developed fever 2 days later so he should be properly defined “presymptomatic”. Most of the symptoms (see Table 1) were statistically associated with a positive nasopharyngeal swab result, showing that clinical history may help to select HCWs to be investigated by means of RT-PCR in case of shortage of diagnostic resources.

We found that close contact (within 2 m), exposure lasting more than 15 min and the use of PPE were not statistically related to the chance of contagion, except for the use of medical masks (*p* < 0.01). These findings raise question about the effectiveness of current ECDC contact classification in estimating the risk of contagion among HCWs who assists patients with COVID-19.

Since most positive HCWs were in contact with an infected colleague, we suppose that the PPE was under-used in this specific context.

In a study carried out in Milan on a large population including 5700 HCWs, the rate of positive nasopharyngeal swab was 10%, whereas the proportion of positive results was only 2.6% through random tested HCWs [12].

The lower proportion of positive HCWs identified in our study might be due both to misclassification of close contacts (some operators might have intentionally over reported the duration of the contact to receive the diagnostic swab) and to the lower incidence rate in the general population of Rome, compared to that of Milan in the same period.

In a large screening for Sars-Cov-2 carried on in a group of HCWs employed in a UK teaching hospital, 3% of asymptomatic subjects tested positive for SARS-CoV-2, whereas 14% of symptomatic tested positive (*p* < 0.0001, Fisher’s exact test) [13].

A possible limitation of our study is that we did not evaluate the possible influence of gender, age and job task in relation to the positive swab result following occupational exposure. The specific pattern of exposure due to the job task could influence the efficiency of Sars-Cov-2 transmission.

In a total of 11,890 specimens collected among HCWs in the Veneto region of Italy, positive results were found in 238 workers, with an incidence of 4.0%. In these HCWs, the risk of infection was not significantly related to gender, age or job task, whereas working area and occupation were predictors of infection [14]. In a previous paper carried out in a similar population among a large hospital population regardless of a specific exposure event, a significant association was found between infection and BMI and night shift work status (OR 3.049, 95%CI 1.260–7.380 and OR 7.15, 95%CI 2.91–17.51 for night shift work and BMI > 30, respectively); the possible role of shift work was not evaluated in the present study [15].

Based on the results of our study, unprotected contacts with COVID-19 subjects (including colleagues), regardless of their exposure risk classification, should self-isolate and contact occupational medicine services in the case of symptoms developing within 14 days after the exposure.

Symptomatic subjects should immediately undergo swab for SARS CoV-2. According to ECDC, the contact HCWs should no longer be considered at risk of developing the infection if still asymptomatic after 14 days from the last exposure.

## 5. Conclusions

Our study shows that the rate of COVID-19 transmission among unprotected HCWs is relatively low, the presence of symptoms being the best predictor of positivity at the nasopharyngeal swab. Based on the result of our study, low-risk contact should be included in post-exposure screening and strictly monitored to evaluate the onset of COVID-like symptoms.

On the basis of our data, the contagion from positive colleagues represents a major cause of nosocomial transmission, raising questions regarding the effectiveness of current prevention measures in the occupational setting. The use of PPE and social distancing measures should also be implemented during the time not spent in direct contact with patients, and the occupational medicine services should implement infection control plans including providing HCWs with information on COVID-19.

## Figures and Tables

**Table 1:** Correlation between case investigation characteristics and results of the nasopharyngeal swab.

		Positive Nasopharyngeal Swab	*p*
Lasting more than 15 min	Yes	7 (1.05%)	0.381
	No	5 (1.47%)	
Distance within 2 m	Yes	9 (1.03%)	0.194
	No	3 (2.31%)	
Use of gloves	Yes	3 (0.84%)	0.330
	No	9 (1.38%)	
Use of masks	Yes	7 (0.92%)	<0.01
	No	5 (2.02%)	
Use of eye protection	Yes	1 (1.75%)	0.505
	No	11 (1.16%)	
Anosmia	Yes	4 (100%)	<0.01
	No	8 (0.80%)	
Asthenia	Yes	6 (15.79%)	<0.01
	No	6 (0.62%)	
Myalgia	Yes	7 (26.93%)	<0.01
	No	5 (0.51%)	
Headache	Yes	5 (5.43%)	<0.01
	No	7 (0.77%)	
Fever	Yes	3 (11.11%)	<0.01
	No	9 (0.92%)	
Cough	Yes	6 (4.00%)	<0.01
	No	6 (0.70%)	
Sore throat	Yes	5 (27.62%)	0.048
	No	7 (0.85%)	
Other Symptoms	Yes	11 (3.22%)	<0.01
	No	1 (0.15%)	
